# Supplementary material for: Optoelectronic analysis of technical factors and performance of elite-level air pistol shooting
Source: PLoS One. 2022 Jan 19;17(1):e0262276. doi: 10.1371/journal.pone.0262276 (PMC8769313; doi:10.1371/journal.pone.0262276)
Supplement: S1 Data — (PDF) [file pone.0262276.s001.pdf]

Date	Sesion	Shoot number	score	time	Hold within 10	Hold within 10.5	Hold within relative 10	Hold within relative 10.5	S1	S2	DA	Right time	Shoot type	S1-S2	Deficit 10	Deficit 10.5	Serie	Shot placement	Score without decimals	Score	Cluster	Hold within 9	Inner ten	DA 250ms
43466	1	1	10,2	5,6	0,59	0,01	0,95	0,72	91	98,5	6,9	1	1	7,5	0,36	0,71	1	1	10		2	100	1	5,1
43466	1	2	10,8	8,9	0,81	0,44	0,86	0,42	118,1	67,8	1,5	2	1	-50,3	0,05	-0,02	1	4	10		1	100	2	1,1
43466	1	3	10,3	9,8	0,69	0,07	0,75	0,2	106,9	89,8	4	2	1	-17,1	0,06	0,13	1	6	10		1	100	1	3,6
43466	1	4	10,7	6,5	0,43	0,08	0,74	0,31	120,3	146,4	8,7	1	1	26,1	0,31	0,23	1	4	10		2	100	2	5,8
43466	1	5	9,9	12,2	0,78	0,25	0,87	0,37	132,1	161,5	9,3	2	2	29,4	0,09	0,12	1	8	9		2	100	1	12,2
43466	1	6	9,8	9,4	0,69	0,15	0,84	0,38	104,1	109,8	7,4	2	2	5,7	0,15	0,23	1	4	9		1	96	1	4
43466	1	7	9,5	9	0,69	0,21	0,83	0,18	116,2	131,3	8,8	2	2	15,1	0,14	-0,03	1	7	9		1	100	1	5,4
43466	1	8	10,2	7,5	0,6	0,3	0,66	0,34	125,5	105,4	7,5	1	1	-20,1	0,06	0,04	1	3	10		2	100	1	3,6
43466	1	9	10,9	4,9	0,35	0,1	0,68	0,16	107,8	153,7	7,3	1	1	45,9	0,33	0,06	1	5	10		2	100	2	5,4
43466	1	10	9,9	9,3	0,64	0,23	0,74	0,18	127	110,9	11,4	2	2	-16,1	0,1	-0,05	1	7	9		1	100	1	6,5
43466	1	11	10,5	11,7	0,53	0,06	0,88	0,41	88,2	128,6	2,8	2	1	40,4	0,35	0,35	2	2	10		2	100	2	3,2
43466	1	12	9,2	7,4	0,7	0,16	0,88	0,43	122,7	195,1	11,1	1	2	72,4	0,18	0,27	2	3	9		2	100	1	7,9
43466	1	13	10,4	5,7	0,76	0,36	0,93	0,38	108,6	90,4	5,9	1	1	-18,2	0,17	0,02	2	2	10		2	100	2	3,4
43466	1	14	9,4	11,1	0,5	0,13	0,79	0,3	133,6	145,6	6,5	2	2	12	0,29	0,17	2	8	9		2	100	1	4,4
43466	1	15	10,4	8,8	0,9	0,28	0,94	0,37	89,1	61,1	5,4	2	1	-28	0,04	0,09	2	1	10		1	100	2	3,6
43466	1	16	9,3	9,8	0	0	0,95	0,22	101,6	116,7	10,5	2	2	15,1	0,95	0,22	2	7	9		1	100	1	6,4
43466	1	17	10,3	5,8	0,8	0,35	0,76	0,45	133,9	124,9	6,2	1	1	-9	-0,04	0,1	2	2	10		2	100	1	3,4
43466	1	18	9,3	10,8	0,5	0,03	0,93	0,44	96,1	81,6	8	2	2	-14,5	0,43	0,41	2	3	9		2	100	1	7,3
43466	1	19	10,8	13,8	0,91	0,24	0,96	0,28	131,6	111,2	2,6	2	1	-20,4	0,05	0,04	2	7	10		2	100	2	2,6
43466	1	20	9,5	7,1	0,14	0	0,52	0,14	120,4	117,5	9,4	1	2	-2,9	0,38	0,14	2	7	9		2	100	1	2,1
43466	1	21	9,3	7,1	0,15	0	0,58	0,1	108,4	97,5	13,3	1	2	-10,9	0,43	0,1	3	4	9		2	100	1	8,2
43466	1	22	9,2	13,5	0,09	0	0,68	0,09	108,5	125,2	3,9	2	2	16,7	0,59	0,09	3	8	9		2	100	1	2,4
43466	1	23	10,6	7,1	0,85	0,38	0,86	0,44	125,7	101,1	3,1	1	1	-24,6	0,01	0,06	3	3	10		2	100	2	6,6
43466	1	24	10,1	8,8	0,34	0,04	0,75	0,31	121,9	80,3	7,9	2	1	-41,6	0,41	0,27	3	9	10		1	100	1	5,4
43466	1	25	10,5	8,3	0,83	0,43	0,93	0,5	104,7	45,3	1,8	2	1	-59,4	0,1	0,07	3	2	10		1	99	2	1,2
43466	1	26	10,4	10,1	0,27	0,06	0,9	0,36	91,6	123,8	6,9	2	1	32,2	0,63	0,3	3	1	10		2	100	2	3,5
43466	1	27	10,3	14,2	0,62	0,24	0,64	0,25	129,7	109,6	8,2	2	1	-20,1	0,02	0,01	3	3	10		2	100	1	2,9
43466	1	28	10,3	7,9	0,54	0,2	0,68	0,32	135,5	153,6	2,1	1	1	18,1	0,14	0,12	3	7	10		2	97	1	4,3
43466	1	29	10	5,3	0,39	0,05	0,87	0,35	113,5	71,8	3,4	1	1	-41,7	0,48	0,3	3	8	10		2	100	1	0,8
43466	1	30	10,6	8,7	0,69	0,41	0,69	0,27	124,3	166,9	3,1	2	1	42,6	0	-0,14	3	8	10		1	100	2	6,8
43466	1	31	9,5	8,4	0,61	0,1	0,96	0,4	101,3	96,4	6,3	2	2	-4,9	0,35	0,3	4	6	9		1	100	1	6,8
43466	1	32	9,8	8,6	0,55	0,05	0,84	0,42	102,4	97	1,7	2	2	-5,4	0,29	0,37	4	2	9		1	100	1	3,9
43466	1	33	10,2	6,6	0,46	0,07	0,84	0,3	108,8	97,2	6,1	1	1	-11,6	0,38	0,23	4	1	10		2	96	1	1,9
43466	1	34	9,1	7,4	0,66	0,32	0,83	0,44	87,1	113,3	11,5	1	2	26,2	0,17	0,12	4	7	9		2	100	1	6,9
43466	1	35	10,6	4,2	0,36	0,21	0,75	0,33	91,6	85,5	8,9	1	1	-6,1	0,39	0,12	4	6	10		2	100	2	2,9
43466	1	36	10,2	7,4	0,84	0,46	0,92	0,68	89,5	76,7	2,1	1	1	-12,8	0,08	0,22	4	2	10		2	100	1	1,7
43466	1	37	10,7	7,2	0,33	0,08	0,93	0,45	100,5	135,3	9,2	1	1	34,8	0,6	0,37	4	3	10		2	100	2	5,6
43466	1	38	10,5	7,2	0,99	0,56	0,99	0,59	104,5	75,7	3,9	1	1	-28,8	0	0,03	4	1	10		2	100	2	4,3
43466	1	39	10,8	8,3	0,66	0,25	0,6	0,23	106,2	89,8	7,3	2	1	-16,4	-0,06	-0,02	4	7	10		1	100	2	4,6
43466	1	40	10,1	9,7	0,93	0,51	0,92	0,59	98,6	133,2	6	2	1	34,6	-0,01	0,08	4	1	10		1	99	1	7,1
43466	1	41	10	8,3	0,41	0,08	0,61	0,28	103,3	66,6	6,4	2	1	-36,7	0,2	0,2	5	4	10		1	100	1	4,3
43466	1	42	9,5	10,5	0,4	0,08	0,69	0,32	106,3	91,1	4,2	2	2	-15,2	0,29	0,24	5	8	9		2	100	1	5,5
43466	1	43	10,3	4,9	0,67	0,08	0,96	0,69	102,4	109,8	0,9	1	1	7,4	0,29	0,61	5	7	10		2	99	1	2,3
43466	1	44	10,8	6,8	0,79	0,4	0,78	0,39	92,2	50,5	1,2	1	1	-41,7	-0,01	-0,01	5	3	10		2	100	2	1,9
43466	1	45	10	4,5	0,56	0,16	0,94	0,46	94,3	101,5	11,7	1	1	7,2	0,38	0,3	5	3	10		2	100	1	9,1
43466	1	46	10,5	5,9	0,42	0,08	0,77	0,31	123,4	133,9	9,3	1	1	10,5	0,35	0,23	5	1	10		2	100	2	8,1
43466	1	47	10,2	7,6	0,21	0	0,91	0,37	99,4	88,4	11	1	1	-11	0,7	0,37	5	9	10		2	100	1	7,7
43466	1	48	10,3	7,9	0,26	0	0,84	0,5	105,1	114,2	10,6	1	1	9,1	0,58	0,5	5	4	10		2	100	1	11,7
43466	1	49	10,6	9,9	0,72	0,29	0,76	0,22	120,7	144,1	5,2	2	1	23,4	0,04	-0,07	5	4	10		1	98	2	1,9
43466	1	50	10,4	11,6	0,52	0,12	0,72	0,33	139	119,7	7,7	2	1	-19,3	0,2	0,21	5	3	10		2	100	2	4,1
43466	1	51	10,1	10,3	0,61	0,06	0,9	0,29	102,5	97,4	3,9	2	1	-5,1	0,29	0,23	6	4	10		2	100	1	0,8
43466	1	52	9,9	5,4	0,64	0,08	0,93	0,45	106,2	112,5	6,9	1	2	6,3	0,29	0,37	6	4	9		2	100	1	6
43466	1	53	9,9	7,4	0,55	0,03	0,84	0,44	113	125,6	6,6	1	2	12,6	0,29	0,41	6	4	9		2	100	1	3,4
43466	1	54	10,5	7	0,42	0,1	0,88	0,19	88,6	98,1	8,4	1	1	9,5	0,46	0,09	6	6	10		2	100	2	5,4
43466	1	55	10	5	0,9	0,21	0,92	0,27	76,7	51,9	7,6	1	1	-24,8	0,02	0,06	6	1	10		2	100	1	2,2
43466	1	56	10,3	7	0,87	0,15	0,9	0,51	87,9	63,2	1,8	1	1	-24,7	0,03	0,36	6	2	10		2	100	1	1
43466	1	57	10	7,2	0,74	0,21	0,76	0,35	117,9	145,3	6,2	1	1	27,4	0,02	0,14	6	1	10		2	100	1	3,8
43466	1	58	10	5,5	0,24	0,02	0,72	0,28	104,5	87,3	6,6	1	1	-17,2	0,48	0,26	6	7	10		2	100	1	5,6
43466	1	59	10,2	4,6	0,67	0,19	0,8	0,16	101,7	90,8	7,7	1	1	-10,9	0,13	-0,03	6	2	10		2	100	1	3,3
43466	1	60	10,9	6,5	0,78	0,24	0,91	0,48	101,8	126,1	4,2	1	1	24,3	0,13	0,24	6	5	10	583	2	97	2	3,7
43586	2	1	9,4	11,3	0,36	0,07	0,8	0,18	102,6	63,2	6,4	2	2	-39,4	0,44	0,11	1	2	9		2	100	1	5,2
43586	2	2	10,3	10,3	0,9	0,27	0,93	0,49	62,4	79,7	5,3	2	1	17,3	0,03	0,22	1	8	10		2	100	1	2,7
43586	2	3	9,5	9,9	0,33	0,02	0,87	0,45	71,8	58	8,5	2	2	-13,8	0,54	0,43	1	1	9		1	100	1	3,1
43586	2	4	10,4	7,9	0,77	0,16	0,96	0,7	78	95,3	1,4	1	1	17,3	0,19	0,54	1	8	10		2	100	2	2,9
43586	2	5	10,7	15,6	0,88	0,29	0,8	0,34	81,7	60,5	2,6	2	1	-21,2	-0,08	0,05	1	2	10		2	100	2	0,8
43586	2	6	10,4	9,8	0,71	0,15	0,69	0,22	97,6	81,9	2,9	2	1	-15,7	-0,02	0,07	1	1	10		1	100	2	3,7
43586	2	7	9,9	9,3	0,31	0	0,99	0,36	84,4	85,7	2,9	2	2	1,3	0,68	0,36	1	1	9		1	100	1	2,2
43586	2	8	10,1	11,7	0,79	0,34	0,84	0,27	96,6	75	6,1	2	1	-21,6	0,05	-0,07	1	8	10		2	100	1	7,3
43586	2	9	10,3	9,1	0,5	0,18	0,94	0,4	67,9	93,2	4,7	2	1	25,3	0,44	0,22	1	8	10		1	100	1	7,4
43586	2	10	10,5	14,9	0,92	0,32	0,97	0,54	82,7	48,6	6,1	2	1	-34,1	0,05	0,22	1	6	10		2	100	2	5,2
43586	2	11	10,3	12	0,84	0,22	0,9	0,36	93,3	102,4	3,3	2	1	9,1	0,06	0,14	2	6	10		2	100	1	5,7
43586	2	12	9,2	13,8	0,1	0	0,93	0,36	111,5	89,3	3,9	2	2	-22,2	0,83	0,36	2	1	9		2	100	1	4,4
43586	2	13	9,6	13,6	0,58	0,13	0,86	0,48	85,3	87	5,2	2	2	1,7	0,28	0,35	2	6	9		2	100	1	2,8
43586	2	14	10,5	13,1	0,68	0,24	0,87	0,42	73,7	92,8	8,6	2	1	19,1	0,19	0,18	2	8	10		2	100	2	6,5
43586	2	15	9,9	9,2	0,89	0,24	0,88	0,26	84,5	84,9	10,3	2	2	0,4	-0,01	0,02	2	1	9		1	100	1	5,7
43586	2	16	10,2	11,6	0,93	0,47	0,93	0,43	89,3	90,2	4,8	2	1	0,9	0	-0,04	2	3	10		2	100	1	1,2
43586	2	17	10,2	11,6	0,55	0,17	0,82	0,43	88,6	65,6	1,5	2	1	-23	0,27	0,26	2	3	10		2	100	1	1,4
43586	2	18	10,4	10,8	0,79	0,18	0,94	0,37	113,1	103,2	2,7	2	1	-9,9	0,15	0,19	2	6	10		2	100	2	4
43586	2	19	10	8,7	0,76	0,45	0,89	0,47	97,7	73,5	4,9	2	1	-24,2	0,13	0,02	2	3	10		1	100	1	2,6
43586	2	20	9,7	12,2	0,42	0,21	0,81	0,27	99	127,3	3,9	2	2	28,3	0,39	0,06	2	2	9		2	100	1	7,2
43586	2	21	9,6	10,3	0,66	0,16	0,63	0,39	89,6	112	8,4	2	2	22,4	-0,03	0,23	3	3	9		2	100	1	1,1
43586	2	22	10,1	5,4	0,59	0,26	0,95	0,33	87,6	73,2	1,8	1	1	-14,4	0,36	0,07	3	9	10		2	100	1	3,5
43586	2	23	9,9	10	0,78	0,21	0,82	0,34	100,4	103,1	11,6	2	2	2,7	0,04	0,13	3	1	9		1	100	1	5,8
43586	2	24	10,1	14,2	0,81	0,34	0,95	0,4	75,4	106,3	2,7	2	1	30,9	0,14	0,06	3	2	10		2	100	1	1,9
43586	2	25	9,8	11,2	0,44	0,12	0,89	0,36	94,5	60,6	3,3	2	2	-33,9	0,45	0,24	3	6	9		2	100	1	0,3
43586	2	26	10,3	8,9	0,83	0,39	0,88	0,35	91,2	97,9	4,2	2	1	6,7	0,05	-0,04	3	6	10		1	100	1	5,3
43586	2	27	10,3	9,6	0,88	0,46	0,88	0,48	76,9	55,3	5,5	2	1	-21,6	0	0,02	3	4	10		1	100	1	4,8
43586	2	28	10,2	14,8	0,6	0,17	0,94	0,24	100,4	109,5	5,1	2	1	9,1	0,34	0,07	3	2	10		2	100	1	5
43586	2	29	10,1	10,2	0,97	0,25	1	0,36	90,4	63,6	4,1	2	1	-26,8	0,03	0,11	3	3	10		2	100	1	3,2
43586	2	30	9,8	12,1	0,61	0,34	0,81	0,28	107,1	167	11,5	2	2	59,9	0,2	-0,06	3	1	9		2	100	1	5,8
43586	2	31	10,3	11,1	0,5	0,06	0,94	0,43	102,4	87,4	5,1	2	1	-15	0,44	0,37	4	3	10		2	100	1	2,9
43586	2	32	9,5	7,9	0,67	0,14	0,72	0,19	84	95,5	11,2	1	2	11,5	0,05	0,05	4	3	9		2	100	1	4,6
43586	2	33	9,6	7,4	0,2	0,08	0,78	0,34	82,3	101,5	1,4	1	2	19,2	0,58	0,26	4	2	9		2	100	1	2,2
43586	2	34	10,8	10	0,89	0,35	1	0,51	87,9	104,5	3	2	1	16,6	0,11	0,16	4	7	10		1	100	2	2,3
43586	2	35	10,9	15,7	0,74	0,45	0,92	0,26	67,9	93	3,8	2	1	25,1	0,18	-0,19	4	5	10		2	100	2	1,7
43586	2	36	9,7	9,7	0,87	0,33	0,87	0,3	97,4	73,5	11,2	2	2	-23,9	0	-0,03	4	3	9		1	100	1	6,6
43586	2	37	10,1	11,8	0,57	0,15	0,67	0,07	94,1	90,1	7,7	2	1	-4	0,1	-0,08	4	8	10		2	100	1	1,5
43586	2	38	10,2	9	0,42	0,19	0,82	0,26	87,3	83,4	10,2	2	1	-3,9	0,4	0,07	4	9	10		1	100	1	5,5
43586	2	39	9,3	10,9	0,06	0	0,93	0,55	83,4	127,5	12,7	2	2	44,1	0,87	0,55	4	3	9		2	100	1	12,6
43586	2	40	10,2	10,5	0,82	0,27	0,9	0,4	101,8	94,5	4,5	2	1	-7,3	0,08	0,13	4	6	10		2	100	1	4,8
43586	2	41	9,7	9,9	0,72	0,33	0,96	0,4	97	85,3	7	2	2	-11,7	0,24	0,07	5	2	9		1	100	1	2,9
43586	2	42	10,9	10,1	1	0,68	1	0,8	70,8	77,4	2	2	1	6,6	0	0,12	5	5	10		2	100	2	2,4
43586	2	43	10,1	7,6	0,84	0,36	0,85	0,47	100,4	157	5,4	1	1	56,6	0,01	0,11	5	1	10		2	100	1	8,1
43586	2	44	10,3	9,2	0,9	0,25	0,96	0,29	106,4	116,3	7	2	1	9,9	0,06	0,04	5	8	10		1	100	1	6,5
43586	2	45	9,7	9,9	0,67	0,35	0,89	0,21	98,7	127,4	5,9	2	2	28,7	0,22	-0,14	5	2	9		1	100	1	4,7
43586	2	46	10,1	6	0,62	0,18	0,62	0,17	106,6	87,1	10,6	1	1	-19,5	0	-0,01	5	2	10		2	100	1	5,3
43586	2	47	10,2	7,9	0,36	0,14	0,78	0,22	76	78,5	12,8	1	1	2,5	0,42	0,08	5	8	10		2	100	1	4,8
43586	2	48	10,6	1	0,79	0,25	0,86	0,45	96,5	46,7	1,1	2	1	-49,8	0,07	0,2	5	1	10		2	100	2	2,1
43586	2	49	9,7	8,7	0,59	0,12	0,97	0,32	57,1	49,6	5	2	2	-7,5	0,38	0,2	5	1	9		1	100	1	3,5
43586	2	50	10,4	12,6	0,76	0,46	0,69	0,34	68,3	65,7	4,7	2	1	-2,6	-0,07	-0,12	5	1	10		2	100	2	4,7
43586	2	51	10,7	11,8	0,66	0,16	1	0,69	66,3	92,5	5,7	2	1	26,2	0,34	0,53	6	4	10		2	100	2	5,8

43586	2	52	10,6	8,2	0,85	0,24	0,92	0,37	81,7	80,2	4,9	2	1	-1,5	0,07	0,13	6	1	10		1	100	2	6,6
43586	2	53	10,2	14,3	0,45	0,05	0,91	0,27	74,7	97,8	9	2	1	23,1	0,46	0,22	6	9	10		2	100	1	8,7
43586	2	54	9,9	10	0,87	0,3	0,88	0,59	88,1	74,8	9,9	2	2	-13,3	0,01	0,29	6	8	9		1	100	1	8,7
43586	2	55	9,9	12,8	0,86	0,36	0,88	0,59	81,3	66,4	6,3	2	2	-14,9	0,02	0,23	6	2	9		2	100	1	3
43586	2	56	10,3	10,3	0,81	0,26	0,88	0,45	94,5	53,3	1,3	2	1	-41,2	0,07	0,19	6	1	10		2	100	1	1
43586	2	57	9,4	9,1	0,77	0,29	0,83	0,24	77,9	96,5	9,9	2	2	18,6	0,06	-0,05	6	3	9		1	100	1	7,6
43586	2	58	10,7	7,8	0,73	0,32	0,78	0,46	91,9	69,6	4,4	1	1	-22,3	0,05	0,14	6	4	10		2	100	2	5,6
43586	2	59	10,6	7,9	0,94	0,42	0,97	0,6	73,1	45,6	4,4	1	1	-27,5	0,03	0,18	6	6	10		2	100	2	5,6
43586	2	60	9,6	9,9	0,6	0,25	0,74	0,26	91,6	79,6	7,1	2	2	-12	0,14	0,01	6	3	9	578	1	100	1	4,3
43557	3	1	9,9	8,3	0,37	0,1	0,95	0,33	111,3	107,6	3,3	2	2	-3,7	0,58	0,23	1	4	9		1	100	1	1,5
43557	3	2	9,2	7,7	0,59	0,22	0,7	0,23	119,1	125,5	10,4	1	2	6,4	0,11	0,01	1	7	9		2	100	1	9
43557	3	3	10,3	7,2	0,47	0,06	0,64	0,39	120,4	168,8	9,8	1	1	48,4	0,17	0,33	1	1	10		2	100	1	9,5
43557	3	4	9,4	9,2	0,53	0,16	0,77	0,18	116,8	119,1	10,8	2	2	2,3	0,24	0,02	1	4	9		1	100	1	11,3
43557	3	5	10,6	8,2	0,45	0,07	0,55	0,19	138,1	151	4	2	1	12,9	0,1	0,12	1	8	10		1	96	2	2,7
43557	3	6	10,3	5,5	0,41	0,06	0,77	0,33	98,9	128,7	3,4	1	1	29,8	0,36	0,27	1	7	10		2	100	1	3,9
43557	3	7	9,7	10,8	0,35	0,06	0,99	0,44	120	98,7	1,9	2	2	-21,3	0,64	0,38	1	7	9		2	100	1	2,5
43557	3	8	9,2	11,7	0,5	0,03	0,86	0,33	138,7	118,2	7,8	2	2	-20,5	0,36	0,3	1	4	9		2	100	1	6,8
43557	3	9	9,1	7,7	0,31	0,06	0,94	0,54	124,7	153,9	7,8	1	2	29,2	0,63	0,48	1	4	9		2	100	1	5,6
43557	3	10	10,1	5	0,58	0,15	0,85	0,43	133,9	194,5	1,5	1	1	60,6	0,27	0,28	1	7	10		2	100	1	3,3
43557	3	11	10,6	10,5	0,25	0,07	0,94	0,57	97,1	119,1	6,5	2	1	22	0,69	0,5	2	1	10		2	100	2	2
43557	3	12	10,4	7,5	0,7	0,24	0,77	0,25	144	149,6	7,3	1	1	5,6	0,07	0,01	2	1	10		2	100	2	3,2
43557	3	13	10,7	10,3	0,8	0,45	0,91	0,36	126,8	73,8	3,3	2	1	-53	0,11	-0,09	2	4	10		2	100	2	1,7
43557	3	14	9,7	7,7	0,79	0,24	0,84	0,34	125,2	132,9	7,8	1	2	7,7	0,05	0,1	2	2	9		2	100	1	6,6
43557	3	15	10,8	6,4	0,65	0,28	1	0,41	84,5	82,1	5,9	1	1	-2,4	0,35	0,13	2	3	10		2	100	2	3,6
43557	3	16	9,7	11,8	0,77	0,39	0,81	0,32	125,9	99,7	7,9	2	2	-26,2	0,04	-0,07	2	9	9		2	100	1	8,6
43557	3	17	10,1	9,3	0,39	0,08	0,78	0,19	108,7	84,6	7,1	2	1	-24,1	0,39	0,11	2	7	10		1	100	1	6,2
43557	3	18	9,5	8,4	0,8	0,35	0,91	0,49	106,6	113,7	8,4	2	2	7,1	0,11	0,14	2	8	9		1	100	1	5,6
43557	3	19	9,8	8,4	0,9	0,41	1	0,67	99,1	108,2	5,4	2	2	9,1	0,1	0,26	2	4	9		1	100	1	5,9
43557	3	20	9,9	6,6	0,68	0,28	0,74	0,31	108,1	84,9	11,3	1	2	-23,2	0,06	0,03	2	2	9		2	100	1	4,9
43557	3	21	10,3	5,8	0,83	0,23	0,86	0,46	108,4	91,5	7,4	1	1	-16,9	0,03	0,23	3	6	10		2	100	1	10
43557	3	22	10,2	7,6	0,88	0,29	0,87	0,37	125,4	129,3	4,9	1	1	3,9	-0,01	0,08	3	1	10		2	100	1	5,6
43557	3	23	10,5	13,7	0,63	0,17	0,91	0,39	93,8	87,6	2,8	2	1	-6,2	0,28	0,22	3	7	10		2	100	2	1,5
43557	3	24	10,1	8,3	0,76	0,34	0,79	0,26	93,8	91,7	7,9	2	1	-2,1	0,03	-0,08	3	3	10		1	100	1	2,3
43557	3	25	10,2	9,1	0,65	0,16	0,76	0,24	129,7	156,9	7,9	2	1	27,2	0,11	0,08	3	4	10		1	100	1	3,2
43557	3	26	9,7	7,5	0,63	0,22	0,93	0,51	95,7	78,1	4,3	1	2	-17,6	0,3	0,29	3	4	9		2	100	1	4,3
43557	3	27	10,1	6,8	0,85	0,38	0,95	0,57	108	100,3	3,2	1	1	-7,7	0,1	0,19	3	1	10		2	100	1	2,4
43557	3	28	10,9	9,2	0,74	0,32	0,83	0,41	116,3	72	4,2	2	1	-44,3	0,09	0,09	3	5	10		1	100	2	3
43557	3	29	9,9	10,9	0,82	0,37	0,86	0,36	126,7	117,7	8,4	2	2	-9	0,04	-0,01	3	9	9		2	100	1	9,6
43557	3	30	9,6	7	0,54	0,11	0,77	0,27	111,7	134	10,6	1	2	22,3	0,23	0,16	3	1	9		2	100	1	7,2
43557	3	31	10,5	12	0,78	0,28	0,83	0,47	101,3	54,8	0,4	2	1	-46,5	0,05	0,19	4	1	10		2	100	2	2,2
43557	3	32	9,4	9,1	0,43	0,08	0,98	0,37	106	107,4	6,6	2	2	1,4	0,55	0,29	4	1	9		1	100	1	8,1
43557	3	33	9,9	9,3	0,77	0,26	0,84	0,19	147,1	132,8	7,6	2	2	-14,3	0,07	-0,07	4	6	9		1	100	1	7,7
43557	3	34	10	6,6	0,61	0,2	0,63	0,3	102,8	164,6	11,5	1	1	61,8	0,02	0,1	4	6	10		2	100	1	4,4
43557	3	35	9,6	5,1	0,76	0,38	0,81	0,33	96,1	106,9	9,1	1	2	10,8	0,05	-0,05	4	4	9		2	100	1	7,1
43557	3	36	10,8	7,9	0,5	0,04	0,97	0,43	96,8	130,6	7,6	1	1	33,8	0,47	0,39	4	4	10		2	100	2	6,5
43557	3	37	9,8	8,5	0,41	0,17	0,45	0,18	107,7	100,7	9,4	2	2	-7	0,04	0,01	4	8	9		1	100	1	1,9
43557	3	38	10	6,7	0,74	0,35	0,82	0,47	94,7	78,7	5	1	1	-16	0,08	0,12	4	7	10		2	100	1	3
43557	3	39	10,2	6,7	0,49	0,11	0,87	0,22	153,6	171	2,2	1	1	17,4	0,38	0,11	4	3	10		2	100	1	3,7
43557	3	40	9,7	8	0,82	0,3	0,85	0,31	115,7	96,2	7,8	1	2	-19,5	0,03	0,01	4	2	9		1	100	1	4
43557	3	41	9,8	9,2	0,63	0,08	0,73	0,41	122,2	95,5	9,4	2	2	-26,7	0,1	0,33	5	4	9		1	100	1	3,3
43557	3	42	10,3	5,7	0,97	0,25	0,96	0,32	88,7	139,6	5,6	1	1	50,9	-0,01	0,07	5	9	10		2	100	1	5,2
43557	3	43	10,5	6	0,59	0,12	0,82	0,24	102,1	74,7	3,7	1	1	-27,4	0,23	0,12	5	9	10		2	100	2	3,4
43557	3	44	9,5	7,1	0,52	0,1	0,68	0,34	104,7	144,8	13,2	1	2	40,1	0,16	0,24	5	6	9		2	100	1	8,2
43557	3	45	10,3	5,6	0,72	0,2	0,77	0,17	115,5	127	4,7	1	1	11,5	0,05	-0,03	5	8	10		2	100	1	3,6
43557	3	46	10	10,4	0,68	0,25	0,78	0,26	112,1	133,5	6,3	2	1	21,4	0,1	0,01	5	3	10		2	100	1	3,7
43557	3	47	10,1	9,2	0,29	0,04	0,77	0,14	91,4	151,2	2,9	2	1	59,8	0,48	0,1	5	1	10		1	100	1	7,2
43557	3	48	10,3	7,3	0,45	0,02	0,99	0,69	89,2	96,1	7,1	1	1	6,9	0,54	0,67	5	9	10		2	100	1	5,9
43557	3	49	10,5	5,7	0,72	0,44	0,77	0,36	103,1	81,3	4,7	1	1	-21,8	0,05	-0,08	5	9	10		2	100	2	0,3
43557	3	50	10	8,5	0,71	0,18	0,96	0,49	77,6	74,7	1,9	2	1	-2,9	0,25	0,31	5	2	10		1	100	1	5,6
43557	3	51	9,9	6,7	0,84	0,24	0,97	0,3	87,5	77,6	6,5	1	2	-9,9	0,13	0,06	6	7	9		2	100	1	5
43557	3	52	10,1	5,7	0,28	0	0,94	0,52	113,9	143,3	6,7	1	1	29,4	0,66	0,52	6	7	10		2	100	1	8,4
43557	3	53	10	6,7	0,46	0,19	0,81	0,3	96,7	139	3,8	1	1	42,3	0,35	0,11	6	4	10		2	100	1	6,3
43557	3	54	10,7	10,9	0,76	0,2	0,89	0,44	122	117,4	6,6	2	1	-4,6	0,13	0,24	6	6	10		2	100	2	3,6
43557	3	55	10	5,3	0,89	0,44	0,94	0,62	79,9	60,9	4,6	1	1	-19	0,05	0,18	6	8	10		2	100	1	4,2
43557	3	56	10,7	7,9	0,58	0,1	0,84	0,35	127,7	160,9	6,7	1	1	33,2	0,26	0,25	6	7	10		2	100	2	4,1
43557	3	57	9,8	6,8	0,81	0,48	0,93	0,38	106,2	76	7,2	1	2	-30,2	0,12	-0,1	6	2	9		2	100	1	2,5
43557	3	58	10,5	13,3	0,45	0,16	0,8	0,32	100,7	114,4	7,3	2	1	13,7	0,35	0,16	6	7	10		2	100	2	0,9
43557	3	59	10,6	10,6	0,81	0,19	0,87	0,44	108,6	132,5	6,9	2	1	23,9	0,06	0,25	6	2	10		2	100	2	3,7
43557	3	60	10,7	7,7	0,61	0,2	0,97	0,51	92,7	79,7	6,9	1	1	-13	0,36	0,31	6	3	10	577	2	100	2	5,4
43680	4	1	10,6	10,2	0,57	0,2	0,82	0,35	148,9	180,8	7,7	2	1	31,9	0,25	0,15	1	6	10		2	100	2	7,6
43680	4	2	10,4	7,8	0,59	0,22	0,96	0,22	112,1	96,8	4,4	1	1	-15,3	0,37	0	1	1	10		2	100	2	7,3
43680	4	3	10,1	8,7	0,73	0,19	0,81	0,37	131,2	73,1	8,3	2	1	-58,1	0,08	0,18	1	6	10		1	100	1	6,8
43680	4	4	9,2	9,1	0,3	0,05	0,31	0,06	106,5	81,4	13,5	2	2	-25,1	0,01	0,01	1	4	9		1	100	1	3,2
43680	4	5	10,4	6,5	0,33	0,08	0,72	0,12	98,3	89,1	9	1	1	-9,2	0,39	0,04	1	9	10		2	100	2	3
43680	4	6	9,7	7,7	0,46	0,18	0,56	0,09	127,1	87,2	16,5	1	2	-39,9	0,1	-0,09	1	3	9		2	96	1	6,8
43680	4	7	10,4	9,9	0,63	0,27	0,79	0,39	153,6	135,8	9,2	2	1	-17,8	0,16	0,12	1	9	10		1	100	2	8,6
43680	4	8	10,8	10,3	0,53	0,11	0,72	0,32	149,4	157,4	5,3	2	1	8	0,19	0,21	1	8	10		2	100	2	1,6
43680	4	9	10,7	5,9	0,78	0,39	0,93	0,44	98,1	120,9	2,1	1	1	22,8	0,15	0,05	1	7	10		2	100	2	2,2
43680	4	10	10,2	8,9	0,83	0,33	0,82	0,4	127,7	154,6	5,8	2	1	26,9	-0,01	0,07	1	1	10		1	100	1	4,1
43680	4	11	10,3	5,7	0,69	0,16	0,87	0,37	129,4	110,7	7,8	1	1	-18,7	0,18	0,21	2	3	10		2	100	1	6,1
43680	4	12	10,5	10,2	0,42	0,08	0,83	0,35	132,3	127,2	7,2	2	1	-5,1	0,41	0,27	2	3	10		2	100	2	5,5
43680	4	13	10,1	9,7	0,75	0,31	0,78	0,25	127,9	90,4	5,5	2	1	-37,5	0,03	-0,06	2	1	10		1	100	1	0,3
43680	4	14	10,2	10,9	0,68	0,29	0,73	0,25	142,4	136,9	3,9	2	1	-5,5	0,05	-0,04	2	1	10		2	100	1	5,4
43680	4	15	9,1	6,3	0,44	0,05	0,75	0,31	117,4	145	8,2	1	2	27,6	0,31	0,26	2	2	9		2	100	1	3,7
43680	4	16	10,4	13	0,51	0,17	0,69	0,17	173,6	175	6,5	2	1	1,4	0,18	0	2	9	10		2	100	2	4,1
43680	4	17	10,3	14,8	0,83	0,11	0,96	0,57	105,5	111,6	8,5	2	1	6,1	0,13	0,46	2	7	10		2	100	1	7,4
43680	4	18	9,9	7	0,57	0,17	0,99	0,38	108,4	103,4	7,2	1	2	-5	0,42	0,21	2	2	9		2	100	1	5,6
43680	4	19	10,8	9,9	0,46	0,19	0,63	0,16	124,7	136,2	5,7	2	1	11,5	0,17	-0,03	2	4	10		1	100	2	0,7
43680	4	20	10,3	8	0,52	0,05	0,79	0,24	130,1	130,2	7,7	1	1	0,1	0,27	0,19	2	9	10		1	100	1	4,6
43680	4	21	10,2	6,7	0,81	0,23	0,84	0,44	121,1	71,8	3,6	1	1	-49,3	0,03	0,21	3	1	10		2	100	1	5,9
43680	4	22	9,9	14,6	0,64	0,32	0,76	0,37	133,5	133,8	12,5	2	2	0,3	0,12	0,05	3	7	9		2	100	1	10,1
43680	4	23	10,3	7,9	0,68	0,2	0,73	0,32	142,9	111,1	5,1	1	1	-31,8	0,05	0,12	3	3	10		2	100	1	3,9
43680	4	24	10,7	6,4	0,38	0,09	0,55	0,12	120,6	154,5	10,4	1	1	33,9	0,17	0,03	3	3	10		2	100	2	8,1
43680	4	25	9,9	10,9	0,7	0,22	0,74	0,24	135,7	147,8	6,1	2	2	12,1	0,04	0,02	3	4	9		2	99	1	6,1
43680	4	26	9	7,7	0,68	0,18	0,83	0,4	112,9	126,9	14,2	1	2	14	0,15	0,22	3	4	9		2	100	1	11,8
43680	4	27	10,3	8,3	0,81	0,24	0,9	0,32	119,9	128,3	7,8	2	1	8,4	0,09	0,08	3	6	10		1	100	1	4,8
43680	4	28	9,2	5,6	0,47	0,02	0,61	0,18	114,8	114	16,3	1	2	-0,8	0,14	0,16	3	1	9		2	97	1	8,5
43680	4	29	10,6	11,1	0,68	0,22	0,68	0,3	121,2	145	3,8	2	1	23,8	0	0,08	3	7	10		2	100	2	5,6
43680	4	30	10,3	6,3	1	0,5	0,98	0,51	98,3	82,8	6,1	1	1	-15,5	-0,02	0,01	3	4	10		2	100	1	7
43680	4	31	9,8	7	0,06	0	0,86	0,42	135,9	132,5	8,3	1	2	-3,4	0,8	0,42	4	9	9		2	100	1	10,4
43680	4	32	8,9	9,7	0,47	0,18	0,77	0,23	104,6	97,6	11,7	2	3	-7	0,3	0,05	4	1	8		1	100	1	4,2
43680	4	33	9,2	6,9	0,74	0,09	0,74	0,09	106,6	112,3	12,1	1	2	5,7	0	0	4	4	9		2	96	1	3,5
43680	4	34	9	6,7	0,02	0	0,89	0,51	108	155	5,2	1	2	47	0,87	0,51	4	9	9		2	100	1	4
43680	4	35	10,8	6,9	0,68	0,18	0,75	0,45	130,9	87,2	3,4	1	1	-43,7	0,07	0,27	4	8	10		2	100	2	2,7
43680	4	36	10,9	5,6	0,35	0,09	0,77	0,28	103,2	115,1	8,3	1	1	11,9	0,42	0,19	4	5	10		2	100	2	5,3
43680	4	37	10,2	13,5	0,44	0,21	0,91	0,26	126,7	115,8	4,5	2	1	-10,9	0,47	0,05	4	4	10		2	100	1	3,9
43680	4	38	9,2	9,3	0,35	0,1	0,68	0,27	153,3	162	6,8	2	2	8,7	0,33	0,17	4	8	9		1	100	1	6,5
43680	4	39	9,8	8,8	0,39	0,17	0,64	0,23	119,5	100,9	2,9	2	2	-18,6	0,25	0,06	4	1	9		1	100	1	3,7
43680	4	40	9,4	13,7	0,55	0,16	0,61	0,29	133,7	161,8	11,8	2	2	28,1	0,06	0,13	4	1	9		2	99	1	6,7
43680	4	41	9,9	5,7	0,01	0	0,87	0,39	98,9	86	3,9	1	2	-12,9	0,86	0,39	5	8	9		2	100	1	3,7
43680	4	42	10	11,7	0,6	0,11	0,74	0,31	153,9	114,7	12	2	1	-39,2	0,14	0,2	5	2	10		2	100	1	10,6
43680	4	43	8,6	11,3	0,51	0,06	0,74	0,35	142	113,9	12,5	2	3	-28,1	0,23	0,29	5	1	8		2	99	1	10,3
43680	4	44	9,9	5,4	0,29	0,06	0,88	0,25	131,6	97,4	7,3	1	2	-34,2	0,59	0,19	5	7	9		2	100	1	9,1
43680	4	45	10,8	7,4	0,72	0,22	0,89	0,53	107,5	81,8	4,3	1	1	-25,7	0,17	0,31	5	3	10		2	100	2	3,1
43680	4	46	10	5,6	0,64	0,16	0,83	0,31	120,3	134,5	12,2	1	1	14,2	0,19	0,15	5	3	10		2	100	1	7,7

43680	4	47	10	7,6	0,45	0,12	0,75	0,2	94,6	69,8	12,2	1	1	-24,8	0,3	0,08	5	9	10		2	100	1	4,2
43680	4	48	9,3	9,9	0,64	0,15	0,61	0,3	127,7	159	11,2	2	2	31,3	-0,03	0,15	5	6	9		1	100	1	8,8
43680	4	49	9,7	11,2	0,63	0,15	0,61	0,15	132,5	138,6	10,5	2	2	6,1	-0,02	0	5	4	9		2	98	1	9
43680	4	50	10,3	7,9	0,81	0,38	0,85	0,39	107,8	142,6	5,1	1	1	34,8	0,04	0,01	5	1	10		2	100	1	2,9
43680	4	51	10,3	8,5	0,6	0,22	0,86	0,26	119	93,8	2,2	2	1	-25,2	0,26	0,04	6	4	10		1	100	1	1
43680	4	52	10,4	10,5	0,81	0,24	0,86	0,39	130,6	222,3	7,6	2	1	91,7	0,05	0,15	6	7	10		2	100	2	8,2
43680	4	53	9,9	9,1	0,7	0,11	0,92	0,55	105,2	75,1	4,7	2	2	-30,1	0,22	0,44	6	1	9		1	100	1	2,9
43680	4	54	10,6	7,6	0,54	0,22	0,88	0,35	109,2	119	6,2	1	1	9,8	0,34	0,13	6	3	10		2	100	2	7,7
43680	4	55	9,7	7,3	0,27	0	0,79	0,24	104,8	102,5	8	1	2	-2,3	0,52	0,24	6	7	9		2	100	1	7,2
43680	4	56	10	10,8	0,81	0,29	0,84	0,34	110,8	99,9	9,8	2	1	-10,9	0,03	0,05	6	3	10		2	100	1	7,3
43680	4	57	10,1	11,8	0,53	0,14	0,82	0,33	109,4	101,3	1,8	2	1	-8,1	0,29	0,19	6	8	10		2	100	1	2
43680	4	58	9,5	8,9	0,75	0,31	0,77	0,3	106,1	112,3	11,4	2	2	6,2	0,02	-0,01	6	9	9		1	100	1	5,2
43680	4	59	10,7	4,8	0,48	0,16	0,75	0,4	110,8	101	4,6	1	1	-9,8	0,27	0,24	6	9	10		2	100	2	4,8
43680	4	60	8,9	9,5	0,57	0,33	0,61	0,1	115,9	126,5	16,6	2	3	10,6	0,04	-0,23	6	2	8	573	1	97	1	15,5
43681	5	1	10,3	7,2	0,38	0,06	0,84	0,3	135,4	118,1	4,2	1	1	-17,3	0,46	0,24	1	2	10		2	100	1	3,2
43681	5	2	10,5	7,4	0,84	0,28	0,84	0,31	143,9	94,5	4,5	1	1	-49,4	0	0,03	1	4	10		2	100	2	5,8
43681	5	3	10	7,5	0,79	0,28	0,75	0,47	113,7	168,8	6,3	1	1	55,1	-0,04	0,19	1	2	10		2	100	1	8,4
43681	5	4	10,2	7,8	0,63	0,2	0,67	0,2	128,2	102,9	9,5	1	1	-25,3	0,04	0	1	9	10		2	100	1	4,8
43681	5	5	10,4	12,1	0,61	0,2	0,72	0,18	118,5	101,2	5,7	2	1	-17,3	0,11	-0,02	1	4	10		2	100	2	4,8
43681	5	6	10	7,2	0,75	0,21	0,84	0,28	143,6	128	4,1	1	1	-15,6	0,09	0,07	1	4	10		2	100	1	6,5
43681	5	7	10,2	9,3	0,7	0,22	0,73	0,31	128	152,2	4,7	2	1	24,2	0,03	0,09	1	8	10		1	100	1	2,3
43681	5	8	10,4	14,8	0,34	0,1	0,44	0,11	138,5	181,4	9	2	1	42,9	0,1	0,01	1	6	10		2	92	2	1,4
43681	5	9	9	7,2	0,53	0,17	0,68	0,14	122,1	120,8	13,8	1	2	-1,3	0,15	-0,03	1	4	9		2	100	1	6,2
43681	5	10	9,3	6,9	0,34	0,14	0,79	0,35	137	133,8	4,9	1	2	-3,2	0,45	0,21	1	8	9		2	100	1	5,1
43681	5	11	10,5	8,2	0,78	0,38	0,78	0,34	136,7	180	4,2	2	1	43,3	0	-0,04	2	9	10		1	100	2	3,7
43681	5	12	10,3	6,3	0,93	0,32	0,99	0,53	101,4	112,5	7,8	1	1	11,1	0,06	0,21	2	3	10		2	100	1	6
43681	5	13	8,3	6,2	0,6	0,19	0,89	0,43	96,3	114,6	17,3	1	3	18,3	0,29	0,24	2	4	8		2	96	1	13,6
43681	5	14	9,5	6,7	0,5	0,06	0,71	0,44	146,9	107,4	12,2	1	2	-39,5	0,21	0,38	2	7	9		2	100	1	12,3
43681	5	15	9,3	5,1	0,53	0,2	0,67	0,18	135,1	112,9	16,2	1	2	-22,2	0,14	-0,02	2	6	9		2	92	1	7,4
43681	5	16	9,7	7,1	0,21	0	0,87	0,49	130,4	149,4	17,9	1	2	19	0,66	0,49	2	6	9		2	97	1	16,4
43681	5	17	10,7	10,3	0,89	0,49	0,91	0,48	119,6	96,7	2,1	2	1	-22,9	0,02	-0,01	2	2	10		2	100	2	1,3
43681	5	18	9,7	13,8	0,47	0,28	0,96	0,22	120,6	83,7	4	2	2	-36,9	0,49	-0,06	2	1	9		2	100	1	7,9
43681	5	19	9,6	12,2	0,46	0,06	0,79	0,42	138,6	222,4	4,3	2	2	83,8	0,33	0,36	2	4	9		2	100	1	4,8
43681	5	20	10,4	8,9	0,47	0,16	0,73	0,35	153,1	161,6	9	2	1	8,5	0,26	0,19	2	3	10		1	95	2	3,5
43681	5	21	9,5	6,1	0,36	0	0,89	0,45	124,2	116,6	3,5	1	2	-7,6	0,53	0,45	3	1	9		2	100	1	4,8
43681	5	22	9,5	11	0,73	0,13	0,83	0,38	148	105,3	7,5	2	2	-42,7	0,1	0,25	3	4	9		2	100	1	5,4
43681	5	23	9,4	6	0,3	0,14	0,66	0,11	133,6	117,3	7	1	2	-16,3	0,36	-0,03	3	9	9		2	100	1	2,9
43681	5	24	9,7	7,6	0,87	0,25	0,91	0,6	88,4	82	8,4	1	2	-6,4	0,04	0,35	3	4	9		2	100	1	6,9
43681	5	25	9,7	12,7	0,45	0,09	0,87	0,36	129,7	166,4	9,6	2	2	36,7	0,42	0,27	3	4	9		2	100	1	9,3
43681	5	26	10,7	6,5	0,38	0,19	0,9	0,44	93,4	81,5	6,1	1	1	-11,9	0,52	0,25	3	7	10		2	100	2	0,9
43681	5	27	9,8	6,6	0,34	0,07	0,51	0,16	95,6	103	13,7	1	2	7,4	0,17	0,09	3	4	9		2	100	1	5
43681	5	28	10,5	8	0,91	0,47	0,89	0,46	123,2	142,8	4,2	1	1	19,6	-0,02	-0,01	3	3	10		1	100	2	5,4
43681	5	29	9,8	6,9	0,13	0	0,56	0,17	122,5	125,8	5,3	1	2	3,3	0,43	0,17	3	8	9		2	100	1	4,7
43681	5	30	10,4	7,4	0,27	0,08	0,78	0,35	132,5	138,5	5,5	1	1	6	0,51	0,27	3	8	10		2	100	2	4
43681	5	31	10	7,8	0,62	0,1	0,64	0,36	141,5	141,4	4,5	1	1	-0,1	0,02	0,26	4	8	10		2	100	1	4,9
43681	5	32	9,8	6,8	0,62	0,25	0,85	0,4	123,6	200	10,4	1	2	76,4	0,23	0,15	4	6	9		2	100	1	8,7
43681	5	33	9,8	9,3	0,51	0,21	0,7	0,22	119,6	113	7,4	2	2	-6,6	0,19	0,01	4	7	9		1	100	1	8
43681	5	34	10,7	6,2	0,67	0,13	0,88	0,35	99,3	100,4	4	1	1	1,1	0,21	0,22	4	7	10		2	100	2	3,2
43681	5	35	9,9	9	0,15	0	0,71	0,21	117,2	160,7	9,7	2	2	43,5	0,56	0,21	4	7	9		1	100	1	5,5
43681	5	36	10,5	6,4	0,46	0,13	0,66	0,31	142,3	159,3	5,1	1	1	17	0,2	0,18	4	8	10		2	100	2	0,5
43681	5	37	9,4	8,4	0,64	0,1	0,96	0,5	116,6	121,2	6,5	2	2	4,6	0,32	0,4	4	7	9		1	100	1	6,6
43681	5	38	10,5	6,9	0,38	0,05	0,7	0,33	120,3	136,7	8,4	1	1	16,4	0,32	0,28	4	8	10		2	100	2	3,9
43681	5	39	10,3	7,2	0,24	0,03	0,66	0,23	116,1	162,2	16,4	1	1	46,1	0,42	0,2	4	1	10		2	98	1	11
43681	5	40	10	7,6	0,55	0,2	0,55	0,17	127,8	115,6	7,7	1	1	-12,2	0	-0,03	4	8	10		2	100	1	1,7
43681	5	41	9,9	8,2	0,5	0,08	0,91	0,47	116,5	96,9	2,5	2	2	-19,6	0,41	0,39	5	8	9		1	100	1	4,2
43681	5	42	9,9	8	0,41	0,01	0,82	0,41	129	120,3	9,7	1	2	-8,7	0,41	0,4	5	3	9		1	100	1	4,1
43681	5	43	10	7,4	0,7	0,38	0,67	0,28	123,7	148,2	5,1	1	1	24,5	-0,03	-0,1	5	2	10		2	97	1	8,4
43681	5	44	10,3	9,3	0,71	0,27	0,78	0,46	155,8	103,1	2,6	2	1	-52,7	0,07	0,19	5	6	10		1	97	1	1,4
43681	5	45	10	6,4	0,45	0,19	0,81	0,26	111,2	115,6	7,1	1	1	4,4	0,36	0,07	5	6	10		2	100	1	2,6
43681	5	46	9,7	5,5	0,7	0,21	0,94	0,41	110	110,4	5,8	1	2	0,4	0,24	0,2	5	8	9		2	100	1	2,8
43681	5	47	9,5	8	0,48	0,1	0,54	0,15	116,4	97,3	16,1	1	2	-19,1	0,06	0,05	5	1	9		1	99	1	9,6
43681	5	48	10,2	6,3	0,92	0,48	0,91	0,54	106,4	84,9	6,9	1	1	-21,5	-0,01	0,06	5	3	10		2	100	1	7
43681	5	49	10,6	6,9	0,81	0,5	0,79	0,41	121,1	107,9	4	1	1	-13,2	-0,02	-0,09	5	3	10		2	97	2	2,7
43681	5	50	10,3	10,1	0,94	0,54	0,95	0,57	105	97,5	4	2	1	-7,5	0,01	0,03	5	1	10		2	100	1	0,9
43681	5	51	10,1	6,6	0,88	0,32	0,84	0,46	117,2	138,6	8,8	1	1	21,4	-0,04	0,14	6	6	10		2	100	1	5
43681	5	52	10,4	7,6	0,74	0,26	0,81	0,27	112,1	59,4	6,4	1	1	-52,7	0,07	0,01	6	3	10		2	98	2	0,7
43681	5	53	10,8	10,3	0,6	0,24	0,94	0,4	93,1	90,9	5,8	2	1	-2,2	0,34	0,16	6	4	10		2	100	2	2,2
43681	5	54	10,5	7,5	0,74	0,28	0,78	0,32	155,7	113,3	5,2	1	1	-42,4	0,04	0,04	6	6	10		2	97	2	4,5
43681	5	55	10,5	6,7	0,92	0,22	0,93	0,33	125,4	112	2,9	1	1	-13,4	0,01	0,11	6	9	10		2	100	2	1,8
43681	5	56	10,7	6,8	0,25	0,12	0,69	0,24	125,2	130,7	12,6	1	1	5,5	0,44	0,12	6	3	10		2	100	2	3,7
43681	5	57	10,7	6,7	0,46	0,13	0,71	0,27	141	124,9	10,3	1	1	-16,1	0,25	0,14	6	3	10		2	100	2	6,2
43681	5	58	10,2	9,3	0,54	0,24	0,77	0,26	98,1	95,7	1,6	2	1	-2,4	0,23	0,02	6	1	10		1	100	1	7
43681	5	59	10,7	8,4	0,83	0,35	0,94	0,5	104,3	94	3,2	2	1	-10,3	0,11	0,15	6	9	10		1	100	2	4,1
43681	5	60	10,5	6,9	0,28	0,02	0,93	0,35	106	142,9	12,2	1	1	36,9	0,65	0,33	6	3	10	576	2	100	1	12,5
43621	6	1	10,7	10,6	0,81	0,22	0,84	0,19	110,8	99,3	3,5	2	1	-11,5	0,03	-0,03	1	8	10		2	100	2	1,5
43621	6	2	9,2	10,8	0,81	0,29	0,89	0,38	103,3	104,6	11,8	2	2	1,3	0,08	0,09	1	9	9		2	100	1	6,9
43621	6	3	10,2	6,3	0,76	0,32	0,92	0,44	112,4	119,9	2,1	1	1	7,5	0,16	0,12	1	2	10		2	100	1	3,3
43621	6	4	10,5	9,8	0,77	0,26	0,94	0,4	120,6	97,2	6,8	2	1	-23,4	0,17	0,14	1	6	10		1	100	2	4,1
43621	6	5	10,2	10,5	0,28	0,01	1	0,67	84,7	117	5,4	2	1	32,3	0,72	0,66	1	3	10		2	100	1	4,7
43621	6	6	10,4	7,1	0,92	0,3	1	0,42	77,6	115,4	1,4	1	1	37,8	0,08	0,12	1	3	10		2	100	2	4,7
43621	6	7	9,5	10,4	0,65	0,25	0,83	0,42	98,5	111	13,4	2	2	12,5	0,18	0,17	1	9	9		2	100	1	7
43621	6	8	10,5	6,8	0,54	0,23	0,89	0,35	145,7	142,3	3,1	1	1	-3,4	0,35	0,12	1	6	10		2	92	2	6,2
43621	6	9	10,3	6,9	0,9	0,61	0,92	0,69	91,1	126,9	4,5	1	1	35,8	0,02	0,08	1	4	10		2	100	1	1,6
43621	6	10	9,4	5,3	0,53	0,17	0,97	0,58	81,5	42,9	6,7	1	2	-38,6	0,44	0,41	1	3	9		2	100	1	3,1
43621	6	11	10,5	10,3	0,66	0,33	0,76	0,27	116,6	140,6	1	2	1	24	0,1	-0,06	2	6	10		2	100	2	6,3
43621	6	12	10,3	11,8	0,58	0,13	0,99	0,54	101,5	112,1	1,7	2	1	10,6	0,41	0,41	2	2	10		2	100	1	1,6
43621	6	13	10,3	9,8	0,67	0,18	0,82	0,32	144,5	121,9	2,2	2	1	-22,6	0,15	0,14	2	8	10		1	96	1	4,8
43621	6	14	10,1	7,2	0,44	0,11	0,85	0,33	99	77	2,6	1	1	-22	0,41	0,22	2	6	10		2	100	1	4,6
43621	6	15	9,6	13,2	0,81	0,32	0,9	0,51	110,8	131,8	7,2	2	2	21	0,09	0,19	2	9	9		2	93	1	6,7
43621	6	16	10,6	1,3	0,73	0,27	0,68	0,23	102,6	149	4	2	1	46,4	-0,05	-0,04	2	2	10		2	97	2	1,4
43621	6	17	9,5	9,3	0,26	0	0,79	0,44	94,6	81,6	9,9	2	2	-13	0,53	0,44	2	1	9		1	100	1	1,9
43621	6	18	10,4	8,6	0,89	0,43	0,98	0,54	92,5	84,9	6,7	2	1	-7,6	0,09	0,11	2	2	10		1	100	2	6,4
43621	6	19	9	8,5	0,36	0,01	0,95	0,28	104,2	88,7	7,6	2	2	-15,5	0,59	0,27	2	2	9		1	100	1	7,3
43621	6	20	10	6	0,85	0,43	0,96	0,4	87,6	100,2	7,6	1	1	12,6	0,11	-0,03	2	8	10		2	95	1	4,2
43621	6	21	10,2	8,2	0,92	0,46	1	0,56	84,4	85	4	2	1	0,6	0,08	0,1	3	3	10		1	100	1	2,2
43621	6	22	10,2	10	0,47	0,13	0,87	0,29	98,5	89,1	2,7	2	1	-9,4	0,4	0,16	3	6	10		1	100	1	4,9
43621	6	23	10,6	6,5	0,32	0,11	0,89	0,39	103,9	81,6	8,4	1	1	-22,3	0,57	0,28	3	9	10		2	100	2	5,8
43621	6	24	9,9	6,5	0,69	0,44	0,86	0,56	88,9	82,6	5,3	1	2	-6,3	0,17	0,12	3	2	9		2	100	1	2,3
43621	6	25	10,4	5,9	0,98	0,51	1	0,55	87,1	71,7	3,5	1	1	-15,4	0,02	0,04	3	2	10		2	100	2	1,4
43621	6	26	10,2	12,4	0,44	0,14	0,78	0,37	112,2	100,8	8,7	2	1	-11,4	0,34	0,23	3	8	10		2	100	1	7,1
43621	6	27	10,4	9,6	0,24	0	0,74	0,31	107,2	92,5	12,5	2	1	-14,7	0,5	0,31	3	7	10		1	100	2	8,3
43621	6	28	9,9	6,9	0,91	0,33	0,89	0,33	87,5	108	7,8	1	2	20,5	-0,02	0	3	2	9		2	100	1	2,6
43621	6	29	9,7	12,1	0,53	0,15	0,91	0,52	120,9	138,2	3,4	2	2	17,3	0,38	0,37	3	3	9		2	100	1	2
43621	6	30	9,3	6,2	0,32	0,19	0,66	0,16	100,2	80,4	9,1	1	2	-19,8	0,34	-0,03	3	1	9		2	100	1	3,1
43621	6	31	10,1	6,8	0,13	0	0,84	0,41	95,8	80,8	8	1	1	-15	0,71	0,41	4	4	10		2	100	1	4,3
43621	6	32	10,5	9,7	0,41	0,02	0,94	0,42	83,3	51,4	6	2	1	-31,9	0,53	0,4	4	4	10		1	100	2	1,4
43621	6	33	10,5	7,3	0,42	0,21	0,93	0,3	102,6	95,8	10,8	1	1	-6,8	0,51	0,09	4	3	10		2	100	2	6,1
43621	6	34	10,4	9,2	0,79	0,29	0,98	0,59	86,2	110	7,5	2	1	23,8	0,19	0,3	4	9	10		1	100	2	7,7
43621	6	35	9,5	3,2	0	0	0,93	0,37	91,2	79,2	8,7	2	2	-12	0,93	0,37	4	8	9		2	100	1	8,2
43621	6	36	10,6	9,2	0,8	0,4	0,8	0,45	92,5	120	3,3	2	1	27,5	0	0,05	4	1	10		1	100	2	3,6
43621	6	37	10,4	7,6	0,68	0,24	0,84	0,43	129,7	82,9	1,1	1	1	-46,8	0,16	0,19	4	1	10		2	100	2	2,6
43621	6	38	10,5	9,3	0,64	0,27	0,64	0,25	112,2	95,9	1,9	2	1	-16,3	0	-0,02	4	3	10		1	100	2	2
43621	6	39	9,8	5,8	0,44	0	0,81	0,37	90,1	114	13,7	1	2	23,9	0,37	0,37	4	1	9		2	98	1	6,1
43621	6	40	10,3	11,2	0,88	0,2	0,9	0,4	94,9	62,4	2,2	2	1	-32,5	0,02	0,2	4	6	10		2	100	1	2,1
43621	6	41	10,7	2,8	0,6	0,16	0,91	0,39	115	155,7	8,1	2	1	40,7	0,31	0,23	5	4	10		2	100	2	6,6

43621	6	42	9,3	9,5	0,4	0,08	0,76	0,15	108,8	100,3	7,5	2	2	-8,5	0,36	0,07	5	2	9		1	100	1	2,5
43621	6	43	10,2	6,1	0,51	0,12	0,74	0,25	121,2	91,3	9,4	1	1	-29,9	0,23	0,13	5	4	10		2	97	1	4,4
43621	6	44	10,5	8,5	0,64	0,14	0,62	0,16	129,4	84,3	0,5	2	1	-45,1	-0,02	0,02	5	4	10		1	97	2	7
43621	6	45	9,6	8,6	0,34	0,1	0,9	0,37	97,3	79,5	5,1	2	2	-17,8	0,56	0,27	5	2	9		1	100	1	2,9
43621	6	46	10,3	6,5	0,81	0,33	0,95	0,62	88,9	117,9	6,6	1	1	29	0,14	0,29	5	1	10		2	100	1	7,3
43621	6	47	9,7	5,7	0,62	0,16	0,9	0,36	99,2	92,7	4,9	1	2	-6,5	0,28	0,2	5	3	9		2	99	1	4,3
43621	6	48	10	7,9	0,98	0,41	0,98	0,61	89,1	95,8	6,5	1	1	6,7	0	0,2	5	4	10		2	100	1	4,7
43621	6	49	10,1	6,7	0,94	0,3	0,95	0,68	92,7	116,2	6,8	1	1	23,5	0,01	0,38	5	6	10		2	97	1	5,8
43621	6	50	9,4	6	0,46	0,12	0,83	0,35	93,7	76,6	6,8	1	2	-17,1	0,37	0,23	5	2	9		2	100	1	3,7
43621	6	51	10,4	7,1	0,34	0,05	0,9	0,55	105,8	54	6,5	1	1	-51,8	0,56	0,5	6	1	10		2	100	2	4,1
43621	6	52	10	9	0,53	0,12	0,67	0,22	114,3	103,3	6,4	2	1	-11	0,14	0,1	6	8	10		1	98	1	2,7
43621	6	53	10,1	5,8	0,96	0,27	1	0,58	84,9	103,1	3,6	1	1	18,2	0,04	0,31	6	3	10		2	100	1	2,4
43621	6	54	10,2	11,4	0,6	0,22	0,82	0,21	89,3	110,2	7,6	2	1	20,9	0,22	-0,01	6	2	10		2	97	1	7,2
43621	6	55	10	11,5	0,55	0,02	0,89	0,41	107,5	86,6	2,7	2	1	-20,9	0,34	0,39	6	7	10		2	100	1	2,2
43621	6	56	10,1	8,1	0,17	0,02	0,78	0,39	92,1	65,4	8,8	2	1	-26,7	0,61	0,37	6	7	10		1	100	1	4,6
43621	6	57	10,3	7,5	0,57	0,14	0,95	0,44	100,6	100,6	2,2	1	1	0	0,38	0,3	6	1	10		2	100	1	2,1
43621	6	58	9,7	9,2	0,32	0,02	0,73	0,32	89,6	102,8	2,2	2	2	13,2	0,41	0,3	6	7	9		1	100	1	3,8
43621	6	59	9,8	9	0,57	0,2	0,92	0,28	97	117	3,3	2	2	20	0,35	0,08	6	4	9		1	100	1	1,9
43621	6	60	10,2	10,4	0,63	0,17	0,91	0,43	109,8	129,7	1,1	2	1	19,9	0,28	0,26	6	7	10	582	2	100	1	1,9
